# In vitro evaluation of osteoblast responses to carbon nanotube-coated titanium surfaces

**DOI:** 10.1186/s40510-016-0136-y

**Published:** 2016-07-27

**Authors:** K. Subramani, S. N. Pandruvada, D. A. Puleo, J. K. Hartsfield, S. S. Huja

**Affiliations:** 1 Division of Orthodontics, University of Kentucky, Lexington, KY USA; 2 Department of Biomedical Engineering, University of Kentucky, Lexington, KY USA

**Keywords:** Multi-walled carbon nanotubes, Titanium, Osteoblast response, Orthodontic mini screw implants, Temporary anchorage devices

## Abstract

**Background:**

The effects of surface roughness and carboxyl functionalization of multi-walled carbon nanotubes (MWCNTs) mixed with collagen coated onto titanium (Ti) substrates on MC3T3-E1 osteoblasts were evaluated.

**Methods:**

The proliferation, differentiation, and matrix mineralization were investigated using (1) smooth-surfaced Ti discs, (2) Ti discs coated with collagen and MWCNT (Ti-MWCNT), and (3) Ti discs coated with collagen and MWCNT-COOH (Ti-MWCNT-COOH) for applications in orthodontic mini screw implants (MSIs). The coatings were uniform when analyzed using scanning electron microscopy (SEM), and surface roughness was evaluated by surface profilometry that demonstrated similar surface roughness (*R*_*a*_, mean ± SD) in the MWCNT (0.83 ± 0.02 μm) and MWCNT-COOH (0.84 ± 0.01 μm) groups. MTT (3-(4,5-dimethylthiazol-2-Yl)-2,5-diphenyltetrazolium bromide) assay was performed after days 1, 3, and 7 to assess proliferation. Alkaline phosphatase (ALP)-specific activity was assessed after day 7 to quantify differentiation. Alizarin red staining was measured after day 28 to quantify matrix mineralization. All data were analyzed with JMP Pro11 software (SAS, USA) with a statistical significance of *p*  < 0.05.

**Results:**

Surface profilometry demonstrated similar surface roughness (*R*_*a*_, mean ± SD) in the MWCNT (0.83 ± 0.02 μm) and MWCNT-COOH (0.84 ± 0.01 μm) groups. On day 7, ALP assay showed that MWCNT-COOH (mean ± SD 0.98 ± 0.26 U/μg of protein) enhanced cell differentiation when compared to the uncoated group (*p* = 0.05). Alizarin red staining after 28 days of cell culture revealed that MWCNT-COOH (mean ± SD 1.5 ± 0.2 OD405) increased (*p* = 0.03) matrix mineralization when compared to the uncoated group (0.9 ± 0.09 OD405).

**Conclusions:**

This study showed that coatings containing MWCNT-COOH (increased hydrophilic surface chemistry) influence osteoblast proliferation, differentiation, and matrix mineralization and should be further studied for applications in orthodontic MSIs.

## Background

In the process of orthodontic treatment, clinicians often utilize some teeth as an anchorage point to move other teeth to facilitate tooth alignment and intercuspation [1]. The initial alignment can occasionally lead to undesirable movement of the anchorage teeth or dental segment. To avoid this anchorage loss, temporary anchorage devices (TADs) can be effectively utilized in clinical orthodontic practice [2]. Mini screw implants (MSIs) are manufactured with smooth titanium surfaces (pure titanium or titanium alloy (Ti-6Al-4 V)) and can be placed in multiple locations within the jaw to optimize the desired tooth movement [3]. It is desirable for the MSI to remain in place during orthodontic tooth movement and to be removed by the orthodontist by unscrewing without the need of a trephine after treatment. Unfortunately, MSIs exhibit a 15 to 17 % failure rate [4–6], which is much higher than for traditional endosseous implants [7].

To effectively address this challenge, implant materials need to be identified that could help maintain a fine balance between MSIs staying in place during treatment but not hinder TAD removal. Nanotechnology has provided new materials that have the potential to be utilized in both orthopedic and dental implant applications. Nanoscale surface modifications of dental implants have been beneficial in improving the degree of osseointegration by increase in surface roughness and modification of surface chemistry. Such nanoscale materials and surface modifications should be explored to overcome this challenge encountered with MSIs in orthodontics. Following the discovery of carbon nanotubes (CNTs) in 1991 by Iijima [8], there has been immense interest in these allotropes of carbon due to their unique physical and chemical properties and potential applications in a wide range of fields; from electronic devices and sensors to biocompatible nanocomposite materials of high strength and low weight. CNTs are cylindrical molecules made entirely of carbon atoms that can be formed from a single graphene sheet resulting in the generation of single-walled CNTs (SWCNTs) or from several graphene sheets resulting in the generation of multi-walled CNTs (MWCNTs).

Interestingly, these cylindrical molecules have several features and dimensions similar to the nanoscale collagen fibers of bone. SWCNTs have an average diameter of 1.5 nm, and their length varies from several hundred nanometers to several micrometers [9]. The diameter of MWCNTs typically ranges between 2 and 25 nm [9]. CNTs have been used in two main areas of bone tissue engineering; for structural and electrical enhancement of polymers and ceramic composites and for nanostructured coatings to improve the bioactivity of implant surfaces [10]. Chemically functionalized CNTs have been shown to be compatible with different types of cells, such as rat hippocampal neurons, osteoblasts from rat osteosarcoma, human neuroblastoma cell line, and primary mouse neurons [11–13].

A cell adhesion study on MWCNTs and MWCNTs chemically functionalized with carboxylic acid groups (-COOH) on Ti discs using mouse fibroblast cells (L929) and human umbilical vein endothelial cells (EAHY926) showed that the MWCNT-COOH group had enhanced cell proliferation, viability, and adhesion due to enhanced wettability; indicating superior cyto-compatibility over MWCNTs [14]. When mouse fibroblasts (L929) were cultured on MWCNTs grown on a silicon substrate, the response to this nanotubular surface was demonstrated as high cell viability and exceptional cell adhesion without any functionalization of the nanotubes [15]. Maturation of human osteoblast-like SaOS-2 cells on MWCNT compact substrate were evaluated using assays for osteonectin, osteopontin, and osteocalcin gene expressions, total protein (TP), and alkaline phosphatase (ALP)-specific activity [16]. The results indicated that the MWCNTs stimulated osteogenic maturation of the osteoblasts.

Another study showed that the human CRL 11372 osteoblast cell line synthesized more ALP and calcium on the surfaces of non-functionalized MWCNTs grown from anodized nanotubular titanium surface than on anodized nanotubular titanium without MWCNTs or an unanodized commercial titanium surface currently used in implant manufacturing. This study concluded that bone growth could possibly be enhanced on currently used titanium implants coated with MWCNTs [17]. In another study, titanium plates were aminated and coated with collagen. Carboxylated MWCNTs (MWCNT-COOH) were coated onto this collagen surface and mouse osteoblasts (MC3T3-E1) were cultured on the nanotubes. The results of this study showed increased cell proliferation and adhesion on the carboxylated MWCNTs [18].

Study of SaOS-2 osteoblastic cells on vertically aligned MWCNT scaffolds without purification or functionalization demonstrated the non-toxicity of MWCNTs by flat spreading and monolayer formation of osteoblasts on MWCNT scaffold surfaces [19]. The MC3T3-E1 osteoblast cell line was used to evaluate the effect of collagen MWCNT composite coating on titanium [20]. This study found that cell proliferation increased with the greater amount of MWCNTs. The higher surface roughness of collagen MWCNT composite coated Ti specimens was considered responsible for the relatively greater extent of cell proliferation, viability, and growth. These studies support that MWCNTs are cyto-compatible and they can be functionalized to influence osteoblast response. The purpose of this in vitro study was to evaluate influence of surface roughness and surface chemistry from MWCNT and MWCNT-COOH coatings on a titanium surface and their effects on osteoblast responses.

## Methods

### Specimen preparation

Smooth-surfaced titanium discs (pure Ti, 15 mm in diameter, 1 mm in thickness; Straumann, Switzerland) were treated with 3-aminopropyltriethoxysilane (Sigma Aldrich, USA) in toluene solution (Fisher Scientific, USA) (10 % *w*/*v*) at 80 °C for 12 h [18]. The aminated Ti discs were then soaked in 0.1 % *w*/*v* collagen solution (Sigma Aldrich, USA) at 4 °C for 3 h, rinsed with deionized water, and desiccated at room temperature. MWCNTs, or carboxylated MWCNTs (NuForm Materials, USA), were dispersed in 1 % *w*/*v* sodium cholate (Sigma Aldrich, USA) aqueous solution to a final concentration of 100 ppm with sonication for 90 min. The CNTs were 20–30 nm in diameter and 100 μm in length. The obtained MWCNT suspensions mixed in collagen (2 ml/dish) were poured onto the above collagen-coated Ti discs and incubated at room temperature for additional 3 h. The following three groups were used in this study: (1) smooth-surfaced titanium (Ti) discs, (2) titanium discs coated with collagen and MWCNT (Ti-MWCNT), and (3) titanium discs coated with collagen and MWCNT-COOH (Ti-MWCNT-COOH). In addition, to evaluate the CNT coatings in cross-section, glass coverslips were used as substrates and coated with the MWCNT and MWCNT-COOH nanotubes.

### Surface analysis of substrates

The surface characteristics of the three sample groups were analyzed using scanning electron microscopy (SEM S-4300 Hitachi, Japan). The roughness (*R*_*a*_) of the samples was measured using a surface profilometer (Taylor-Hobson Surtronic 3P, USA). To evaluate the coatings in cross-section, the MWCNT and MWCNT-COOH collagen mixture were coated on glass coverslips and the cross-section was evaluated using SEM.

### Cell culture

Mouse osteoblastic cells (MC3T3-E1), obtained from ATCC, were subcultured and seeded on to different substrates detailed above at a density of 10,000 cells/well in a 24-well plate. The cell cultures were maintained in αMEM (Sigma Aldrich, USA) with 10 % FBS (Sigma Aldrich, USA) and PS antibiotic mixture (Life Technologies, USA) at 37 °C in a humidified atmosphere with 5 % CO_2_ in air.

### SEM analysis of cells cultured on substrates

Changes in the osteoblastic adhesion and morphology were observed using a Bio-LV-scanning electron microscope (SN-3000 Hitachi, Japan). For cell culture, MC3T3-E1 cells were aliquoted at a cell density of 10,000 cells/well and then incubated for 2 days, followed by removal of the medium and washing with PBS. The specimens were fixed in 2.5 % glutaraldehyde and 1 % osmium tetroxide at 4 °C for 2 h, and then sequentially dehydrated using ethanol gradient solutions (50, 60, 70, 80, 90, and 100 %) for 15 min each, followed by drying. The specimens were coated with platinum under Argon gas using a plasma sputtering system (Emscope SC500 K, UK) and then imaged (×2000).

### MTT assay

MTT (3-(4,5-dimethylthiazol-2-Yl)-2,5-diphenyltetrazolium bromide) assay is the most commonly used assay to evaluate cell proliferation and viability [21]. Proliferating cells express higher metabolic activity, converting MTT into a purple-colored formazan product with an absorbance maximum near 570 nm. For this assay, cells were cultured as described above (section cell culture) for a period of 1, 3, and 7 days (*n* = 5/time point). Standard MTT cell proliferation assay [22] was performed for the three Ti substrates. Tissue culture plastic was used as control. The optical density was measured at absorbance = 570 nm using a spectrophotometer (SpectraMax5, Molecular Devices, USA).

### ALP-specific activity

ALP, a noncollagenous protein, is a marker of early osteoblastic differentiation [23]. An ALP assay has been utilized in numerous in vitro studies to evaluate the differentiation of MC3T3-E1 cells [24, 25]. The cells were cultured for a period of 7 days (*n* = 3/group). After the culture period, the medium was removed, and the specimens were washed with p-nitrophenyl phosphate (p-NPP) buffer; 200 μl of diluted Triton X-100 (0.2 % in 1× assay buffer) was aliquoted onto the specimens and sonicated and then centrifuged at 2500 × g at 4 °C for 15 min. Fifty microliters of the supernatant was mixed with 50 μl of ALP substrate solution and then incubated at 37 °C for 30 min, followed by measurement of absorbance (OD405nm) [26]. The total protein content was measured using the Bradford method, and ALP activity was normalized to total protein content.

### Alizarin red staining

Alizarin red staining (ARS) is used to detect and quantify calcium within the deposited mineral matrix [27–29]. For this assay, the cells were cultured on the 5 replicates for a period of 28 days. Alizarin red staining was quantified using standard acetic acid extraction method [30]. The aliquots (150 μl) of the supernatant were measured at absorbance (OD405nm) in 96-well format using opaque-walled, transparent-bottomed plates (Corning, USA) in a spectrophotometer (SpectraMax5, Molecular Devices, USA).

### Statistical analysis

All data were analyzed with JMP Pro11 software (SAS, USA) with a statistical significance of *p*  < 0.05. The Shapiro-Wilk W test was used to assess normality. All the data groups for comparison were normally distributed, so a one-way ANOVA was conducted to evaluate if there was statistically significant difference among the groups. If ANOVA results were significant, the differences among the groups were analyzed by the Tukey-Kramer HSD procedure. For ALP analysis, the Kruskal-Wallis test was performed (n=3/group).

## Results

### Surface analysis

**Fig. 1:**
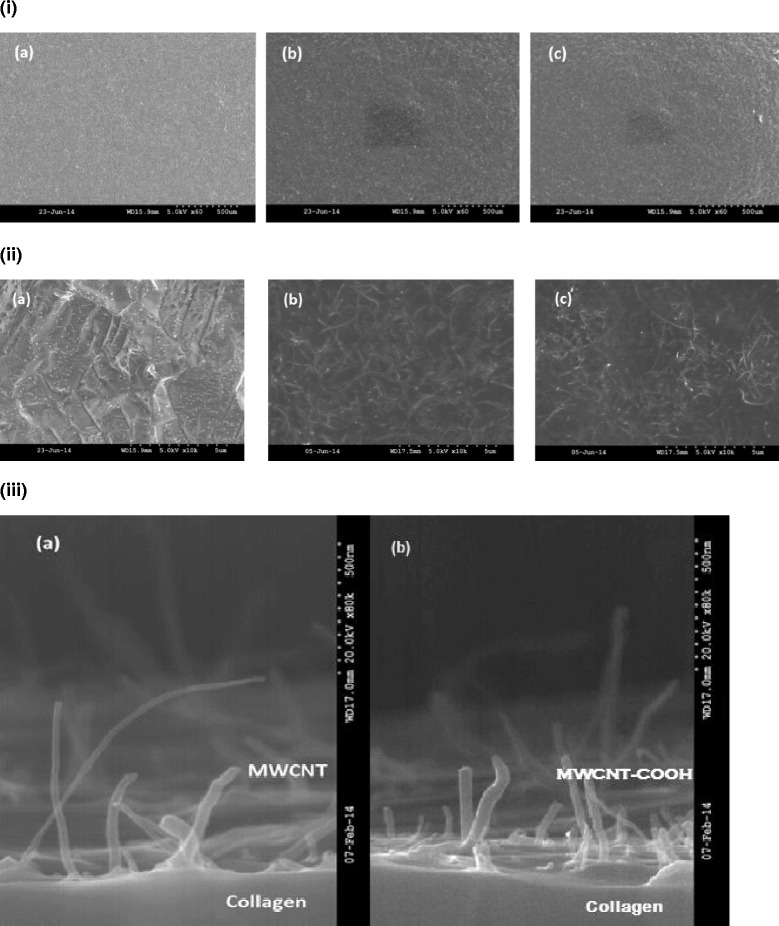
SEM images of titanium discs. **i** SEM images of (*a*) uncoated Ti, (*b*) MWCNT-coated Ti, and (*c*) MWCNT-COOH-coated Ti at ×60 magnification. The CNT coatings are uniform throughout the discs. **ii** SEM images of (*a*) uncoated Ti, (*b*) MWCNT-coated Ti, and (*c*) MWCNT-COOH-coated Ti at ×10,000 magnification. Images show a rougher uncoated Ti surface [*R*_*a*_ = 0.16 ± 0.004 μm (mean ± SD)] and CNTs embedded in the collagen matrix. The *R*_*a*_ for MWCNT and MWCNT-COOH-coated groups (mean ± SD) was 0.83 ± 0.02 and 0.84 ± 0.01 μm, respectively. **iii** SEM image of cross-section of (*a*) MWCNT coating at ×80,000 magnification and (*b*) MWCNT-COOH at ×120,000 magnification on glass coverslip. CNTs can be seen projecting out from the collagen matrix. The CNTs were 20–30 nm in diameter and 100 μm in length

**Fig. 2:**
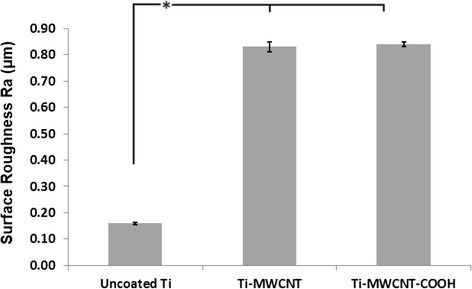
Surface profilometry of titanium discs (n=5/group). *R*_*a*_ (mean ± SD) of the uncoated Ti discs was 0.16 ± 0.004 μm. *R*_*a*_ (mean ± SE) for MWCNT- and MWCNT-COOH-coated groups was 0.83 ± 0.02 and 0.84 ± 0.01 μm, respectively. *Both MWCNT- and MWCNT-COOH-coated Ti discs were significantly different (*p* < 0. 0001) from uncoated Ti discs

The uncoated Ti discs when imaged using SEM demonstrated a relatively smooth surface [Fig. 1i (*a*) and Fig. 1ii (*a*)]. In the MWCNT- and MWCNT-COOH-coated groups, the CNTs were embedded in the collagen matrix [Fig. 1i (*b*, *c*) and 1ii (*b*, *c*)]. The CNT coatings were observed to be mostly uniform on coated discs. The CNTs were observed to be projecting out from the collagen matrix in cross-section of glass coverslips (Figure 1iii (*a*, *b*)). The surface roughness (*R*_*a*_) of the uncoated Ti discs was 0.16 ± 0.004 μm (mean ± SD). The *R*_*a*_ for MWCNT- and MWCNT-COOH-coated groups (mean ± SD) was 0.83 ± 0.02 and 0.84 ± 0.01 μm, respectively (Fig. 2). Both MWCNT- and MWCNT-COOH-coated Ti discs were significantly different (*p* < 0.0001) from uncoated Ti discs.

### SEM analysis of cell morphology

**Fig. 3:**
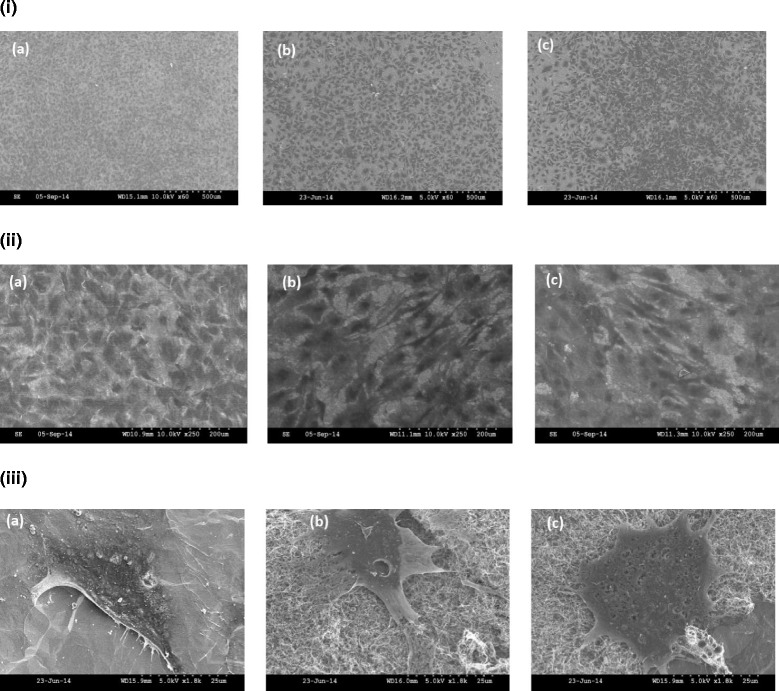
SEM images of MC3T3-E1 cells on titanium discs. **i** SEM images of MC3T3-E1/pre-osteoblast cells on (*a*) uncoated Ti, (*b*) MWCNT-coated Ti, and (*c*) MWCNT-COOH-coated Ti at ×60 magnification. Pre-osteoblasts have adhered to all three substrates after 48 h of cell culture. **ii** SEM images of MC3T3-E1 cells on titanium discs at ×250 magnification. Osteoblasts exhibit typical adherent cell morphology in all substrates. **iii** SEM images of MC3T3-E1 cells on (*a*) uncoated Ti, (*b*) MWCNT-coated Ti, and (*c*) MWCNT-COOH-coated Ti at ×18,000 magnification. Note that the osteoblast adhered on all substrates. In *b* and *c*, the MWCNT and MWCNT-COOH coatings can be observed with the osteoblasts well spread out in both the groups

SEM observation after 48 h of cell culture showed that the osteoblasts adhered in all three groups [Fig. 3i and ii (*a* through *c*)]. The osteoblasts exhibited typical adherent cell morphology in both the MWCNT and MWCNT-COOH groups [Fig. 3iii (*b*, *c*)]. A similar cell morphology was observed in the uncoated group as seen in Fig. 3iii (*a*).

### MTT assay

**Fig. 4:**
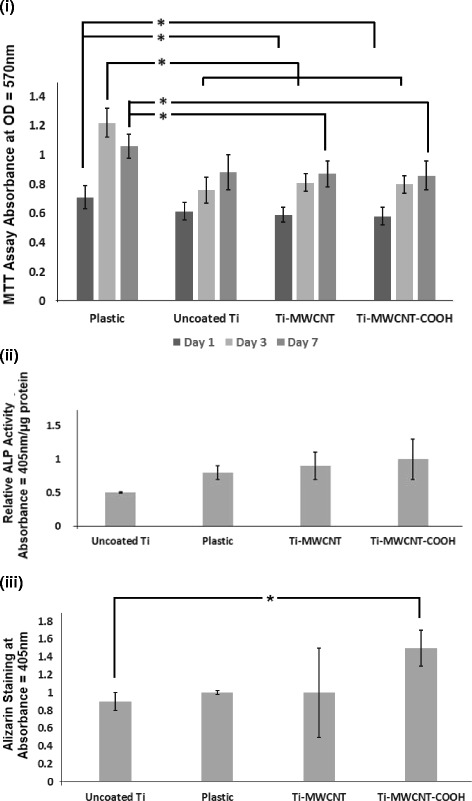
Biocompatibility and mineralization assays on coated titanium discs. **i** MTT assay to assess proliferation of MC3T3-E1 cells cultured on plastic (control), uncoated Ti, MWCNT-COOH-coated Ti, and MWCNT-coated Ti after days 1, 3, and 7 (*n* = 5/group). *Cell proliferation on the plastic group was different from both MWCNT- and MWCNT-COOH-coated Ti discs (*p* = 0.03 and *p* = 0.02, respectively) on day 1. After day 3, cell proliferation on plastic group was significantly different (*p* < 0.0001) from each of the other three groups. After day 7, cell proliferation on the plastic group was significantly different (*p* = 0.03) with both MWCNT- and MWCNT-COOH-coated Ti discs. On day 1, plastic shows the highest amount of cell proliferation (OD570 mean ± SD) (0.71 ± 0.08) followed by uncoated (0.62 ± 0.06), MWCNT group (0.59 ± 0.05), and MWCNT-COOH group (0.58 ± 0.06). On day 3, plastic shows the highest amount of cell proliferation (1.22 ± 0.10) followed by MWCNT group (0.81 ± 0.06), MWCNT-COOH group (0.80 ± 0.06), and uncoated Ti group (0.76 ± 0.09). On day 7, plastic shows the highest amount of cell proliferation (mean ± SE) (1.06 ± 0.08) followed by uncoated Ti group (0.88 ± 0.12), MWCNT group (0.87 ± 0.09) and MWCNT-COOH group (0.86 ± 0.10). **ii** ALP activity of MC3T3-E1 cells cultured on plastic (control), uncoated Ti, MWCNT-COOH-coated Ti, and MWCNT-coated Ti after day 7 (*n* = 3/group). Mean ± SD U/μg protein for the MWCNT-COOH group was 1.0 ± 0.3, the MWCNT group (0.9 ± 0.2), the plastic group (0.8 ± 0.1), and uncoated Ti group (0.5 ± 0.01). There was no statistical significance observed among the groups using the Kruskal-Wallis test (*p*=0.08). **iii** Alizarin red staining assay of MC3T3-E1 cells cultured on plastic (control), uncoated Ti, MWCNT-COOH-coated Ti, and MWCNT-coated Ti after day 28 (*n* = 5/group). Matrix mineralization (mean ± SD) was highest in the MWCNT-COOH group (1.5 ± 0.2) followed by MWCNT (1.0 ± 0.5), plastic (1.00 ± 0.02), and uncoated Ti group (0.9 ± 0.1). Matrix mineralization on the uncoated group was significantly different (*p* = 0.03) from the MWCNT-COOH group

After day 1 of cell culture (Fig. 4i), the optical density (cell proliferation) (mean ± SD) for MTT was the highest in the plastic group (0.71 ± 0.08) followed by the uncoated Ti group (0.62 ± 0.06), MWCNT group (0.59 ± 0.05), and MWCNT-COOH group (0.58 ± 0.06). The plastic group was different with from both MWCNT- and MWCNT-COOH-coated Ti discs on day 1 (*p* = 0.03 and *p* = 0.02, respectively). After day 3 of cell culture, the optical density (mean ± SD) was the highest in the plastic group (1.22 ± 0.10) followed by the MWCNT group (0.81 ± 0.06), MWCNT-COOH group (0.80 ± 0.06), and uncoated Ti group (0.76 ± 0.09). The plastic group was significantly different (*p* < 0.0001) from each of the other three groups. The optical density (mean ± SD) was the highest at day 7 in the plastic group (1.06 ± 0.08), followed by uncoated Ti group (0.89 ± 0.12), MWCNT group (0.87 ± 0.09), and MWCNT-COOH group (0.86 ± 0.10). The plastic group was significantly different (*p* = 0.03) from both the MWCNT- and MWCNT-COOH-coated Ti discs. In the plastic group, there was an increase in cell proliferation from day 1 to 3 (*p* < 0.001) and from day 3 to 7 (*p* = 0.03). In the uncoated group, there was an increase in cell proliferation only from day 1 to 7 (*p* < 0.001), but not from day 1 to 3, or day 3 to 7. For the MWCNT and MWCNT-COOH groups, there was an increase from day 1 to day 3 (*p* = 0.0009 and *p* = 0.002, respectively) but not from day 3 to day 7.

### ALP assay

On day 7, the ALP-specific activity (mean ± SD U/μg protein) for the MWCNT-COOH group was 1.0 ± 0.3, the MWCNT group (0.9 ± 0.2), the plastic group (0.8 ± 0.1), and uncoated Ti group (0.5 ± 0.01) as represented in Fig. 4ii. There was no statistical significance observed among the groups using the Kruskal-Wallis test (*p*=0.08). 

### Alizarin red staining

At day 28, matrix mineralization (mean ± SD) was the highest in the MWCNT-COOH group (1.5 ± 0.2) followed by MWCNT (1.0 ± 0.5), plastic (1.00 ± 0.02), and uncoated Ti group (0.9 ± 0.1) as represented in Fig. 4iii. Matrix mineralization on the uncoated group was significantly different (*p* = 0.03) from the MWCNT-COOH group.

## Discussion

Biological response of osteoblasts to an implant substrate is influenced by both surface roughness and surface chemistry [31]. Relatively rougher surfaces have been reported to be more advantageous for osteoblast proliferation, differentiation, and formation of bone matrix since rough surface substrates have more surface area than smoother surface substrates and the osteoblasts adhere to rougher surfaces more than smoother surfaces [32]. Numerous studies have found MWCNTs to be cyto-compatible [11–13, 15–19]. These studies were conducted on neuronal cells [11, 13], osteoblasts [12], and fibroblasts [15] from human and rat sources. When the MWCNTs were functionalized, the presence of COOH groups enhances cell proliferation, viability, and adhesion due to the -COOH groups rendering the surface hydrophilic and wettable [14]. Carboxyl functionalized CNTs (CNT–COOH) have been used as an effective template to chemically synthesize HAp [33]. This is due to the capability of carboxylate ions (COO^–^) to adsorb calcium ions (Ca^2+^) and contribute to HAp crystallization as a result of exposure to phosphate ions (PO_4_^3^). Initiation of HAp nucleation was shown to take place within carboxyl group [34]. Thus, the presence of carboxyl groups on MWCNTs facilitated HAp formation and mineralization.

In this study, MWCNT and MWCNT-COOH were selected to evaluate their effect on MC3T3-E1 osteoblast-like cell line. It was expected that the surface roughness of the MWCNT-coated discs would influence cell proliferation, differentiation, and matrix mineralization; and there would be a difference between these two groups due to the presence or absence of -COOH functional groups. MC3T3 is an osteoblast precursor cell line derived from mouse calvaria [35]. The MC3T3-E1 sub-line is a physiologically relevant cell line for study of transcriptional control in calvarial osteoblasts, which is an appropriate and relevant osteoblast model for in vitro studies and similar to human osteoblasts in terms of proliferation, differentiation, and matrix mineralization [36]. Hence, this cell line was chosen for this in vitro study.

The MC3T3-E1 cells follow a two-stage developmental process including a 1–2-week initiation phase during which cells slowly proliferate, express ALP activity and other bone specific genes, resulting in the formation of collagen matrix [37]. During the second maturation phase of MC3T3-E1 cells occurring from weeks 2–4, matrix mineralization is observed [38, 39]. Matrix mineralization is considered a functional in vitro endpoint reflecting advanced cell differentiation. Interpreting from the MTT, ALP and ARS assays, it can be understood that the presence of MWCNTs in both CNT groups influenced the rate of cell proliferation from day 3 to 7. In a recently published study, which evaluated the effect of MWCNTs coatings deposited on titanium discs on osteoblast growth, it was shown that there was a strong dependence of the extent of osteoblast proliferation and differentiation on the presence of MWCNTs in the coatings [20]. It can be inferred from this study [20] that higher surface roughness due to MWCNTs was responsible for the relatively higher extent of MC3T3-E1 cell proliferation and differentiation.

CRL-11372 osteoblasts synthesized more alkaline phosphatase and mineralized matrix on the surfaces of MWCNTs grown from anodized nanotubular Ti than on anodized nanotubular Ti without MWCNTs [17]. MWCNTs have also been used as additives to enhance the structural properties of biocompatible scaffolds. When MWCNTs were mixed with poly(lactic-co-glycolic acid) (PLGA) microsphere scaffolds, it was shown that the addition of MWCNT made the PLGA scaffold mechanically stronger and elicited enhanced cellular responses from MC3T3-E1 cells in terms of cell proliferation, differentiation and mineralized matrix formation [40].

The cross-section of the coatings showed that there was no topographical difference as both the MWCNTs were similar in dimension and exhibited same surface roughness. The results from our study confirm that the presence of MWCNTs influenced proliferation from day 3 to 7 as suggested by these studies [17, 20, 40]. However, it was lower than the tissue culture plastic group. Tissue culture plastic which is surface treated elicited higher cell proliferation and the presence of MWCNTs as foreign bodies could have influenced the amount of cell proliferation initially. When cell differentiation and matrix mineralization were evaluated after days 7 and 28, the MWCNT-COOH group showed increased cell differentiation and matrix mineralization, when compared to the MWCNT group. The reason for this could be that the presence of -COOH groups influenced differentiation and mineralization. As discussed earlier, COOH group promotes matrix mineralization. The presence of -COOH groups likely increased differentiation on day 7 [14] and matrix mineralization after day 28 due to increased hydrophilicity, surface wettability of MWCNTs and facilitating mineral matrix deposition [33, 34].

The results of our study concur with previously published literature [14, 17, 20, 33, 34, 40]. MWCNT and MWCNT-COOH coatings can be utilized for future in vivo studies to evaluate the effect of these coatings on titanium MSI osseointegration. One of the limitations of our study is the use of collagen to coat CNTs onto Ti discs and no collagen coating on the uncoated Ti group. However, in a recent study to evaluate the effect on MC3T3-E1 growth from collagen MWCNT composite coating deposited on Ti, the control groups included Ti discs coated with only collagen and MWCNTs as controls [20]. Although this study did not use MWCNT-COOH coatings, the results suggested a strong dependence of the extent of cell proliferation, differentiation, and mineralization on the amount of MWCNTs incorporated in the composite compared to the control groups. Another limitation was the limited number of samples (n=3/group) in ALP assay and the data was analyzed taking this limitation into consideration. In an in vivo setting, it is unknown whether a similar result can be seen from the coatings as an animal model is a living-three dimensional system. Host factors (local and systemic) could influence the outcome (adhesion, proliferation, differentiation, and bone formation). However, these coatings on implants should to be evaluated for bone to MSI contact, inflammatory response, and bone remodeling and for reverse torque to unscrew the MSIs; the CNT coating which can influence sufficient initial osseointegration. It is unknown if they will hamper the removal of MSIs or what effect they would have on the interfacial bond strength.

## Conclusions

It can be concluded that MWCNT and MWCNT-COOH coatings on titanium substrates can be engineered to enhance osteoblast response and bone matrix formation in vitro. The presence of -COOH groups enhanced osteoblast proliferation, differentiation, and matrix mineralization on Ti substrates and demonstrate the potential for further investigations.
